# Telemonitoring via Self-Report and Video Review in Community Palliative Care: A Case Report

**DOI:** 10.3390/healthcare5030051

**Published:** 2017-08-31

**Authors:** Deidre D. Morgan, Kate Swetenham, Timothy H. M. To, David C. Currow, Jennifer J. Tieman

**Affiliations:** 1 Palliative and Supportive Services, College of Nursing and Health Sciences, Flinders University, Bedford Park, Adelaide 5001, Australia; Kate.Swetenham@sa.gov.au (K.S.); Timothy.To@sa.gov.au (T.H.M.T.); David.Currow@sa.gov.au (D.C.C.); Jennifer.Tieman@flinders.edu.au (J.J.T.); 2 Southern Adelaide Palliative Services, Daw Park, Adelaide 5041, Australia

**Keywords:** telemonitoring, palliative care, video-conference, community, cancer

## Abstract

Continuous monitoring and management of a person’s symptoms and performance status are critical for the delivery of effective palliative care. This monitoring occurs routinely in inpatient settings; however, such close evaluation in the community has remained elusive. Patient self-reporting using telehealth offers opportunities to identify symptom escalation and functional decline in real time, and facilitate timely proactive management. We report the case of a 57-year-old man with advanced non-small cell lung cancer who participated in a telehealth trial run by a community palliative care service. This gentleman was able to complete self-reporting of function and symptoms via iPad although at times he was reticent to do so. Self-reporting was perceived as a means to communicate his clinical needs without being a bother to the community palliative care team. He also participated in a videoconference with clinical staff from the community palliative care service and his General Practitioner. Videoconferencing with the nurse and GP was highly valued as an effective way to communicate and also because it eliminated the need for travel. This case report provides important information about the feasibility and acceptability of palliative care telehealth as a way to better manage clinical care in a community setting.

## 1. Introduction

Continuous monitoring and management of a person’s symptoms and performance status are vital for the delivery of effective palliative care. This monitoring occurs routinely in inpatient palliative care settings and is informed by clinical observations, patient self-report and the use of standardised clinical assessments. However, such close evaluation in the community has remained elusive. Patient self-reporting using telehealth offers opportunities to identify symptom escalation and functional decline in real time, and facilitate timely proactive management.

We report the case of a 57-year-old man with advanced non-small cell lung cancer who participated in a telehealth trial run by a community palliative care service. Further description and outcomes from the trial have been published elsewhere [1,2,3]. Participants in this trial were invited to self-report his symptoms and performance status using an application on an iPad. There was also opportunity for videoconferencing with both clinical staff from the community palliative care service, along with his General Practitioner.

Willingness of patients with advanced disease to self-report symptoms has been demonstrated in several studies. Successful modes of symptom self-report have included the use of pain diaries and digital pens [4] and mobile phone technology [5]. In addition to self-report of symptoms, current palliative care telehealth research identifies videoconferencing between clinicians about patient care as a key area of focus [6,7]. More recent studies have begun to explore videoconferencing between patients and clinicians [8,9,10]. This case was selected for discussion as the patient and his wife actively participated in all components of the study. It also demonstrates the feasibility and acceptability of a telehealth supported GP case conference, a novel clinical interaction. Both the patient and his wife also expressed a desire for their experiences to inform future patient care.

## 2. Materials and Methods

Participants for this telehealth study were identified from the active client list of a community palliative care service. This community palliative care service cares for adult patients (18 years and over) with a life limiting illness. Study participants ranged in age from 49 to 91 years and self-reported varying levels of familiarity and comfort with the use of telehealth to support their clinical care. Those meeting the inclusion criteria were approached to participate by palliative care community nurses. Consented participants were provided with an iPad and portable wireless internet access and applications developed specifically for the trial. Use of study-provided devices ensured all participants had access to hardware and also ensured consistent technical support when required. In areas where broadband was available and participants were willing, homes were wired up for broadband access, which has a much faster connection speed than cable.

Participants were asked to self-report their symptoms using the Symptom Assessment Scale (SAS) [11] rating symptoms such as pain, breathlessness, fatigue and bowel problems with a numerical score between 0–10 (0 = no distress–10 = worst possible distress). A free text option enabled participants to note other symptoms or concerns not listed in the Symptom Assessment Scale. Participants also self-reported on their performance status (function) using the Australian Karnofsky Performance Status (AKPS) scale [12]. Self-report of function and symptoms was completed via applications on the iPad. If a participant felt too unwell to self-report, caregivers such as a family member or nurse, could enter the participant’s verbally reported score on their behalf.

Patients or carers could not initiate a telehealth consultation. Predetermined SAS and AKPS thresholds triggered email alerts to participants’ palliative care community nurse. Videoconferencing reviews with nursing staff were conducted in response to these alerts and also used as an alternative to a face-to-face home-visit when deemed clinically appropriate. There was also the option for a video-case conference with the General Practitioner (GP) when a participant’s performance status dropped to a predetermined AKPS score of 70 (i.e., 70 = able to care for self but not able to continue with normal activity or work). This threshold was informed by earlier research that found a single GP case conference with community patients (AKPS < 70) resulted in a performance status being maintained for longer and was correlated with fewer hospitalisations [13]. Participants were advised of the thresholds for email alerts; however, they were also advised to contact the palliative care service directly if they had urgent care needs. Participant care needs took priority over study protocol. Video-conferencing enabled linking of people in three locations—the patient at home, the GP in their surgery and the nurse at the palliative care service. Carers completed a Carer Assessment Questionnaire on a weekly or as needed basis. When caregivers’ numerical rating of their needs reached a predetermined threshold, this triggered an email alert to a carer support worker who followed up using videoconferencing, phone or face-to-face as required [14]. This study was approved by the Southern Adelaide Clinical Human Research Ethics Committee, Australia (HREC/13/SAC/88 168.13).

## 3. Results

Paul was a 57-year-old man diagnosed with Stage 4 metastatic non-small cell lung cancer. He lived with his wife, Annette, and extended family (four generations) approximately 45 minutes drive from the nearest hospice. On admission to the palliative care community service, Paul reported worsening pain and weakness in his upper limbs. He had a bilateral cervical lymphadenopathy but no shortness of breath. Paul and his wife both rated his AKPS at 70 at this point in time (i.e., able to manage self-care but unable to complete other activities or work). Paul entered his AKPS and SAS scores routinely over the course of the study. Annette also entered scores as a proxy on Paul’s behalf when he was unable to self-report as well as completing her own carer needs scores.

### 3.1. Underpinned by Clinical Relationships

While self-reporting of symptoms provided an additional means of communicating with the community palliative care team, Paul and Annette did not want to abuse this. They were concerned about bothering nursing staff, reticent to take up their valuable time, and believed an iPad entry would be less disruptive for busy nurses than a phone call. Paul considered capacity to write free text an informative and effective way of communicating care needs in a manner that afforded nurses an opportunity to attend to it when they were able.

“…Just your thoughts and all that. They have got the time. It is like sending text message isn’t it. You don’t have to answer that text message, people do I know, but ‘okay I will come back to that later’, you know what I mean.”

Annette acted as a proxy reporter on Paul’s behalf several times when he was too unwell. However, she said she was concerned about over-dramatising Paul’s symptoms as they both understood that certain symptom scores triggered immediate alerts to the community nurse. Underpinning this couples’ comfort with self-reporting was their earlier experience of the palliative care service’s timely responsiveness to Paul’s previous care needs. This was in marked contrast to his experiences with other areas of the health service. This underscores the importance of clinical relationships as an integral part of a telehealth service. Importantly, Paul noted that if he experienced a crisis such as rapidly escalating pain, he would probably ring rather than rely on the iPad for any urgent communication and care needs.

### 3.2. Accuracy and Clarity

Paul had previously worked as a security guard and stated he placed great importance on clear communication. He noted his computer familiarity enhanced his confidence in his ability to manage the iPad and willingness to use it for clinical care. However, while Paul was comfortable to self-report on his symptoms and function most of the time, there were days when he found it onerous. Some days the visual reminder of numerically rating his symptoms and function was incredibly confronting.

“You just don’t want to know about anything, you just don’t want to answer questions and then the next three days you are feeling on top of the world and okay, let’s take it on.”

Accurate reporting of his symptoms was very important for Paul and Annette. However, Paul’s symptoms fluctuated markedly in intensity and quality and he wanted capacity to give them more than a numerical rating via the iPad. He wanted the opportunity to describe the quality and nature of his symptoms, but also to convey the emotional impact of his symptoms to the community nurses.

“I think like, if there was a note there or just something that… you could say oh, ‘My pain threshold is actually being caused by my fingers [peripheral neuropathy] and I am really concerned about this.’”

While there was capacity to write a few free text words on the SAS tool, Paul wanted an additional section where he could record an electronic summary of his week for the palliative care team because “… you just want to let people know what you are actually going through”. It was important to Paul that the palliative care service understood and actively managed his physical symptoms, but that they also took into consideration how he and Annette were managing overall. The iPad functioned as a screening tool but did not replace human connection.

### 3.3. Immediacy, Connection and Ease

A distinctive feature of this study was its use of videoconferencing to address symptom management issues and also to facilitate collaboration between clinicians involved in client care. A General Practioner case conference was conducted via iPads in three different locations: Paul in his home, the General Practitioner in his surgery and the palliative care nurse in his office. Paul was particularly interested in observing how the General Practitioner and nurse interacted with each other about his care, as well as directly with him. The immediacy of response and on-screen collaboration between the General Practitioner and nurse was welcomed by Paul and Annette. The clinicians’ ability to connect with each other and with Paul and Annette not only strengthened the professional clinical relationship but contributed to Paul feeling he was valued as a person. Paul noted that when he self-reported his symptoms, a “talk fest via conference” could follow, during which time he could raise issues that were concerning to him.

Paul: So they asked each other questions pertaining to me! …They were able to confirm with each other and concur with each other the best way of going about it which was positive…

Interviewer: How does that make you feel?

Paul: Safe.

Annette: And a bond there.

Paul: There is a bond there.

Annette: Somehow.

Paul: But, but it makes me feel, one less worry… I am a person, I don’t think anybody is giving a damn and all that you know. But they do, I know they do, I can see they do.

The ability to observe clinician collaborations was profound for Paul. It heightened his confidence about his current and future care, something he regarded as vital given his ever increasing reliance on the health service. A videoconference allowed him and Annette to “bring up any concerns and have those conerns answered by the right people.” Paul talked about a past history of depression and feeling uncared for during that time. Participating in the teleconference as an active participant and also as an observer, affirmed Paul’s perceived value as a person and went some way to ameliorating a sense of aloneness.

“Having a teleconference helps you feel positive… I felt as if I was a person…the actual conference was all about me, my sickness, my wellbeing.”

For Annette, the videoconference enabled her to direct queries simultaneously to the community nurse and the General Practitioner, who then liaised onscreen and arrived at a plan to manage troublesome symptoms. She described feeling safe as she observed their discussions and expressed relief at having medication issues sorted on the spot.

Both Paul and Annette experienced the videoconference as more personal than a phone call. Annette remarked that from her perspective “…the information and attentiveness is exactly the same as going to the doctor’s surgery, face-to-face.” She noted that the main difference between a face-to-face meeting with the General Practitioner and a teleconference was the absence of a physical examination. Although a physical examination was not possible, the video facility enabled Paul to show the General Practitioner and nurse the physical changes occurring in his body from his bedroom.

“I can show him what is wrong with me. I can show them the nodes, like I did today… These have gotten bigger again.”

Importantly for Paul, video-conferencing reduced his need to travel, easing the substantial symptom burden associated with attending outpatient appointments. He found travel to and from clinics and long waiting times to see doctors exhausting and expensive.

“Pain… and just the nauseousness … the travelling saps you… In home is good. And it is more cost effective too…to go up and down to the hospital…is adding $15.00. Do that three or four times a week…”

Paul had been the primary earner for his family and now unable to work, he and Annette were acutely aware of escalating health costs and impact on the daily life of the four generations living together. Ability to video conference instead of a physical trip in the car relieved burden associated with medical care.

## 4. Discussion

While findings from this case report cannot be generalised, they highlight several important issues. They demonstrate how patients and carers can engage in telehealth supported palliative care and the empowerment enabled by this additional form of communication with the health service. Paul and Annette highly valued the opportunity to play an active role in their care via telehealth. Self-reporting symptoms enhanced their feelings of connection with palliative care, albeit remotely, and provided them with sense of some control. While similar discussions could be held face-to-face with clinicians, Paul wanted to stay in his home for as long as possible and as discussed later, video reviews could prevent a trip to hospital as well as replicating a face-to-face interaction. This telehealth model supported needs-based care by empowering the patient and carer to alert clinicians when their health status/circumstances changed, enabling clinicians to respond in a targeted way to care needs.

Paul and Annette were approached to participate in this study soon after admission to the palliative care service. Clinical contact had been established so there was an existing relationship, however, it was still in its infancy. Timely clinician responsiveness to email alerts flagging changing care needs and an ongoing openess to discuss how the technology was impacting their clinical care served to strengthen clinical relationships with this couple. Clear communication of contingency plans for management of care needs in the event of a technology malfunction and follow up via phone call on occasions when the internet went down enhanced this trust.

This couple made the point of not wanting to overburden the community care team with unnecessary self-reports of changes in symptoms and function. They felt that the text-like capacity of free text responses was not demanding on nurses and enabled clinicians to attend to their needs when it was most convenient. They noted they understood the nurses were also seeing other patients and acknowledged that their own care needs also fitted around other patients’ clinical care. While patient or proxy self-reporting enhanced the community teams’ understanding of real-time changes in symptom and function, interpretation of remotely entered numerical scores and free text comments must be done within the context of an ongoing clinical relationship. It is important to note that each person and their caregivers experience and respond to symptom changes in individual ways. Recognition of changes in patterns of patient and carer self-reporting is perhaps more important than frequency of self-reporting or numerical score of a symptom. The video conferencing component of this study was as important as an opportunity and the ability to talk “face-to-face”. It also conveyed to this couple that they were more than the sum of their symptom, function or caregiver needs scores. Remote monitoring via telehealth did not replace normal clinical care but augmented clinician understanding of patient needs in a way that was not possible before.

As supported by other studies, people receiving community palliative care are receptive to and able to use telehealth and video-conferencing as an adjunct to face-to-face care [8,15,16,17]. However, when patients have encountered difficulties with videoconferencing, willingness and confidence to continue using this mode of communication declines [16,18]. Paul and Annette’s familiarity with computers and ability to manage applications influenced their willingness to participate. This was enhanced by prompt backup from IT support during the study and an established trust and clinical relationship with palliative care staff.

Dedicated IT support is essential to delivery of effective telehealth care. However, the cost of iPads, internet connectivity, application development, and IT support for this study was grant-funded and not part of routine service delivery. As ongoing IT support was not available through the health service, the trial was not continued. Future applications of this technology need to consider ongoing costs for participants. As technology is rapidly becoming an integral part of everyday life, future telehealth programs may adopt a Bring Your Own Device (BYOD) approach. Health service IT infrastructure also needs to consider expansion of services beyond support of internal staff needs to include telehealth services that may incorporate a BYOD program with patients in the community. Timeliness of response to issues with technology influencing care may influence patient willingness to cease or continue use it [17]. Videoconferencing facilitated a “personal” and intimate connection with the palliative care team and General Practitioner, and enabled care needs to be assessed and managed with minimum imposition on Paul and Annette.

A key point of difference in this study was the ability to link up people in up to three locations to participate in the case conference, reducing clinician and patient need to travel. Travelling to see health professionals comes at a financial, physical and emotional cost for patients with advanced disease and their carers. Notably, symptom escalation and burden associated with travel (e.g., pain, fatigue, anxiety) was eliminated by this videoconference review. While case-conferences with community palliative care patients have been found to reduce hospitalisations and enable people to remain at home for longer [13], the burden associated with patient travel for care has received little examination. Although telehealth-supported case-conferences have the potential to further reduce burden and costs associated with delivery of clinical services, they are still not a routine part of care. As noted earlier, dedicated IT support is an essential enabler for telehealth to become a reliable part of routine care. While Paul and Annette had a positive experience with their videoconference, not all participants in this study embraced it as warmly and not all General Practitioners were willing to engage in this type of video-consultation.

There was the potential for bias in the self-reporting of symptoms. In another study, self-reporting of symptoms by patients with worsening heart failure was found to decline over time [19]. As noted in this case study, Paul and Annette were concerned about unnecessarily burdening staff, potentially leading to under-reporting of care needs. Other factors may contribute to under-reporting and warrant consideration. Paul noted that some days he found self-reporting too confronting as it reminded him of his deterioration and pending death. On other days, he simply wanted to spend time with his family and did not want to self-report. While Paul noted he did not report symptoms consistently, other carers in this study like Annette, were able to report symptoms on the patient’s behalf and routinely did so.

This case report also demonstrates that people view participation in research at this time of their lives as a legacy for people who come after them [20,21]. Paul and Annette noted that several aspects of the telehealth program needed refining. However, they reiterated that they hoped learnings gained from their telehealth experiences would inform the future care of those living remotely from palliative care services. 

## 5. Conclusions

While Paul and Annette’s experiences do not represent all participant experiences in this study, nor can they be generalised to a palliative care population as a whole, they do speak to the feasibility and acceptability of telehealth in palliative care. The potential to minimise burden (symptom, financial and emotional) associated with travelling is clear. In this case report, telehealth supported care was an effective adjunct to routine clinical care but did not replace face-to-face care.
